# Chemsex Practices in PrEP: Beyond Addiction and Risk Toward a Healthy Sex Life—Baseline Experiences from a Hospital-Based PrEP Program in Barcelona, Spain

**DOI:** 10.1007/s10461-022-03730-5

**Published:** 2022-06-22

**Authors:** Lorena De La Mora, Ainoa Ugarte, Maria Martínez-Rebollar, Elisa De Lazzari, David García-Hernández, Guillermo Font, Nicolás De Loredo, Estela Solbes, Laia Miquel, Jordi Blanch, Berta Torres, Josep Riera, Iván Chivite, Juan Ambrosioni, Alexy Inciarte, Ana González-Cordón, Esteban Martínez, José Luis Blanco, Josep Mallolas, Montserrat Laguno

**Affiliations:** 1 HIV Unit, Infectious Diseases Service, Hospital Clinic, IDIBAPS, University of Barcelona, Villarroel 170, 08036 Barcelona, Spain; 2 Addiction Unit (GRAC). Psychiatry and Psychology Department, Hospital Clinic of Barcelona, IDIBAPS, CIBERSAM, Barcelona University, Barcelona, Spain; 3 Dermatology Service, Hospital Clinic, IDIBAPS, University of Barcelona, Barcelona, Spain

**Keywords:** Chemsex, PrEP, Sex life, GBMSM

## Abstract

Pre-exposure prophylaxis (PrEP) is a biomedical intervention that has demonstrated efficacy in HIV prevention in individuals at high-risk, among them chemsex users. Out of 190 PrEP users followed at Hospital Clinic of Barcelona until October 2020, 89% reported drug use, and 63% disclosed that they had engaged in chemsex practices, initiated in 64% of cases within the past year. Twenty-one percent used 3 or more drugs simultaneously, being GHB/GBL, nitrites, sildenafil, and methamphetamine the most prevalent combination. Eight percent reported slamming. Forty-one percent described having had negative experiences and 8% did not remember the last time they had sober sex. Methamphetamine, mephedrone, GHB/GBL, and having had open relationships, group sex, double penetration, and fisting were significantly more prevalent. Forty-nine percent admitted being worried about chemsex use, and 18% said they needed help. A comprehensive, interdisciplinary approach is mandatory to enable the attainment of a healthy approach to one’s sex life.

## Resumen

La PrEP es una intervención biomédica eficaz en la prevención del VIH en personas con alto riesgo, entre ellas las personas que practican chemsex. De los 190 usuarios de PrEP seguidos en el Hospital Clínic de Barcelona hasta octubre de 2020, el 89% refirió utilizar drogas y el 63% en contexto de chemsex, iniciando el consumo el 64% durante el último año. El 21% refería policonsumo, siendo GHB/GBL, nitritos, sildenafilo y metanfetamina la combinación más prevalente. El 8% reportó slamming. El 41% describió haber tenido experiencias negativas y el 8% no recordaba la última vez que tuvo sexo sobrio. Metanfetamina, mefedrona, GHB/GBL y haber tenido relaciones abiertas, sexo en grupo, doble penetración y fisting fueron significativamente más frecuentes. El 49% refirió estar preocupado por la práctica de chemsex y el 18% necesitar ayuda. Un abordaje integral e interdisciplinar mejoraría el acompañamiento global de la sexualidad en estas personas.

## Introduction

Pre-exposure prophylaxis (PrEP) is an intervention based on the use of antiretroviral drugs (ART) aimed at preventing human immunodeficiency virus (HIV) infection in seronegative individuals at high risk of infection. The ART drugs used in PrEP include Tenofovir disoproxil fumarate 300 mg-emtricitabine at 200 mg (TDF-FTC) taken once daily, and is the most widely studied combination [1, 2]. Since 2010, multiple clinical trials have shown the efficacy of oral PrEP for the prevention of HIV infection, reducing HIV transmission by up to 86% (90% CI 64–96), with a number-needed-to-treat (NNT) of 13 users in a year if taken as prescribed [3, 4]. In 2014, UNAIDS recommended using PrEP as an additional tool to help end the HIV epidemic. In October 2019, Spain’s National Health System approved the prescription of PrEP [5, 6].

Different groups at high-risk for HIV infection have been studied, with chemsex users being a group of special interest. Chemsex is the intentional use of particular non-prescription drugs to facilitate, enhance, and prolong sexual encounters between gay, bisexual, and other men who have sex with men (GBMSM). Chemsex is distinctive in its intention, time factor, and use of new technologies, and is a subset of sexualized substance use [7]. This phenomenon has emerged in recent years in modern gay culture around the world, and has had an impact on sexual pleasure and sexual health in the community [8]. People engaged in chemsex are more likely to disclose higher-risk sexual practices. Consequently, these users’ HIV and STI probability increases [9–14].

Chemsex is a dynamic phenomenon that has cultural, geographic, and temporal variations; its prevalence differs according to the subgroup in which it is analyzed and due to the heterogeneity in definitions given by different scholars [15, 16]. In the latest European internet survey on MSM (EMIS 2017), results from Spain showed that 14.1% of the respondents had consumed drugs for the purpose of having sexual relations in the past 12 months, with 0.8% reporting injected drug use. This rate was higher among men living with HIV, men born outside of Spain, and cities with populations larger than 500,000 inhabitants [17].

Global information on chemsex practices in PrEP users is limited [18–20], including data from Spain [17, 21].

Our main objective is to describe chemsex practices in PrEP users of the Hospital Clínic of Barcelona (HCB) at the time of their inclusion in the program, and to identify the users’ profiles and specific needs, to better understand de phenomenon in our area and design tailored strategies.

## Methods

### Design

In this descriptive, cross-sectional study we examined a series of individuals who attended a baseline visit for the PrEP program at HCB, Spain between November 2019 and October 2020. Candidates were referred from post-exposure prophylaxis (PEP) and STI consultations; from a local non-governmental organization (NGO), StopSIDA; or came of their own choice after attending briefings given at the hospital. The study was evaluated and accepted by the Ethics Committee of the HCB. (HCB/2020/1197).

### Inclusion and Exclusion Criteria

We included users who met the following criteria in the PrEP program: people with an HIV-positive partner and multiples partners; people or couples with multiple partners; candidates who do not use condoms; those diagnosed with a recent bacterial STI; sex workers or those who inject drugs; and people who have received post-exposure prophylaxis (PEP) or who have already received PrEP. All patients who agreed to participate in the PrEP program signed an informed consent form so that the data could be used for research purposes. The exclusion criteria for taking PrEP were positive serology for HIV and/or hepatitis B virus (HBV), chronic kidney injury (glomerular filtration < 60 mL/min), having an allergy to any of the TDF/FTC components, and a positive pregnancy test.

We defined chemsex practices as the use of drugs in a sexual context, taken intentionally to prolong and enhance one’s sexual experience.

### Variables

We assessed epidemiological characteristics according to the information obtained from the clinical history and from two self-report questionnaires:

Demographic data included gender, age, place of birth, and education level. We obtained sexually transmitted infection (STI) data from rapid HIV tests, triple STI PCR samples (urine, pharynges, and rectum), and serologies (hepatitis A virus [HAV], HBV, hepatitis C virus [HCV], syphilis, and HIV).

In relation to sexual practices, we recorded the number of sexual partners in the last 3 months, locatable sexual partners, group sex practices, and kinds of sex practiced (oral, anal, vaginal, double penetration, fisting, sex toy use, and condom use).

Substance use data included types of drugs, number of drugs consumed at the same time, route of drug use (inhaled, snorted, slamming, sublingual, rectal), drug use time (last month, last 6 months, in the past year, or more than a year ago), whether they had practiced group sex, why they ended a session, whether they had had a negative experience, the last time they had had sex without drugs, individual perceptions of problematic consumption (or not), and the need for help.

### Statistical Analysis

Qualitative variables are described as frequencies and percentages; we compared them between groups using the chi-squared test, reporting the test (Chi-2) and p values, and Fischer’s exact test, reporting the p value. We summarized the quantitative variables using the mean and standard deviation (SD) or the median and interquartile range (IQR); we compared them between groups with the t-test or the Wilcoxon rank sum test. We performed network coincidence analysis based on the multidimensional scaling (MDS) algorithm to examine the combined use of different drugs. We represented the results graphically using the MDS network diagram, where drugs are denoted by circles with sizes that are directly proportional to their frequency; they are connected by three different patterns of lines according to the three levels of significance of the standardized residuals (derived from the frequency table): dotted line = probable coincidence (0.5); dashed line = statistically probable (< 0.05); and solid line = statistically probable (< 0.01). The most preferred combinations of drugs are represented with solid lines, followed by those with dashed lines and finally dotted lines. In the center of the diagram, there are the drugs with the highest number of matches, while less correlated drugs tend to be located on the fringes of the space. In addition, correlated drugs tend to be closer together in space. All tests were two-tailed with a significance level set at < 0.05. We collected data in an electronic case report form (eCRF) implemented in REDCap hosted at the hospital’s clinic. We used the statistical software Stata for data management and statistical analysis (StataCorp. 2019. Stata: Release 16. Statistical Software. College Station, TX: StataCorp LLC.)

## Results

### Basics Results from the Global Cohort

In the global cohort, 190 PrEP users were included, specifically 177 men and 13 women, of which 11 were transgender. One patient was excluded after testing positive for HIV. There was a mean age of 35 (SD 8), and the most common country of origin was Spain at 50% (n = 89), followed by Central-South America (n = 63, 35%). Regarding education level, 70% (n = 109) of the respondents had attended university. Referring to STIs, 31% (n/N = 56/181) of the triple PCR sample tested positive at any testing location, and according to serology testing, 9% (n/N = 17/189) tested positive for syphilis (VDRL) and 1% (n/N = 1/189) for HCV with negative HCV RNA. General characteristics of the overall cohort have already been published [22].

### Drug Consumption Results and Chemsex Practices

**Fig. 1:**
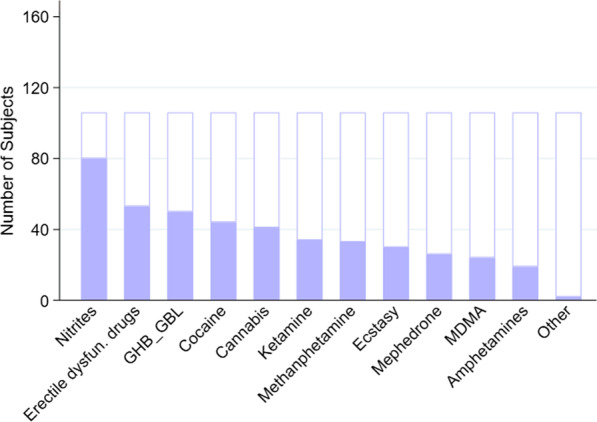
Drug use in a sexual context

A total of 169 PrEP users (89% of the total cohort) disclosed having taken drugs, 106/169 (63%) said they had engaged in chemsex practices, and 21% disclosed chemsex practices in the past month (n/N = 20/96). Thirty-six percent had practiced chemsex for more than a year (n/N = 35/96). We can observe the usage prevalence of each substance in Fig. 1.

We noted polydrug consumption (the use of 3 or more drugs at the same time) in 21% of the 103 participants, while 68% of them combined drugs with alcohol.

**Fig. 2:**
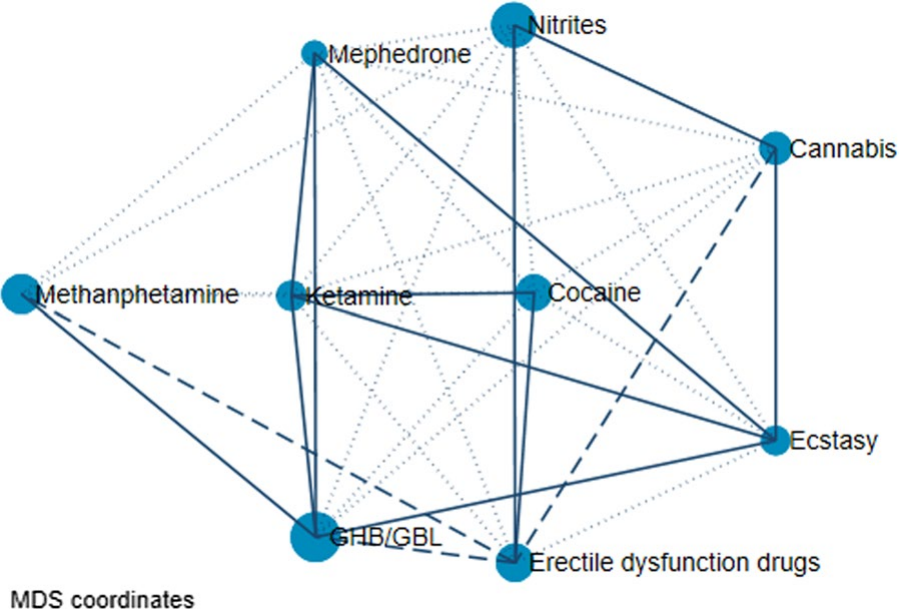
All of the drug combinations (multidimensional scaling network graph). This plot represents the combinations of drugs. The size of each circle represents the incidence of drug use (i.e., the frequency). Three different patterns of lines connecting the drugs (continuous, dashed, and dotted) are defined according to the three levels of significance of the standardized residuals (derived from the frequency tables): dotted line = probable coincidence (0.5); dashed line = statistically probable (< 0.05); solid line = statistically probable (< 0.01). The most preferred combinations of drugs are those represented with solid lines, followed by those with dashed lines and finally dotted lines. In the MDS (multidimensional scaling) network graph, two correlated drugs tend to be closer together. In addition, the drugs with the highest number of matches tend to be located in the center of the diagram. As a result, less correlated events tend to be located on the fringes of the space

Figure 2 represents all drug combinations that have been used by chemsex users, specifically drug use prevalence along with the probability of being combined. The drugs most commonly used in combinations were GHB/GBL, nitrites, methamphetamine, and erectile dysfunction drugs followed by cocaine, cannabis, ketamine, ecstasy and mephedrone. The location in the central part of the network of cocaine and ketamine in Fig. 2 showed that those who consumed these drugs often also consumed other ones, although the majority of those combinations were not the most significant, except for cocaine with sildenafil and ketamine, or ketamine with mephredone and GHB/GBL. GHB/GBL was also used in combination with methamphetamine and erectile dysfunction drugs and separately with ecstasy. Sildenafil and cannabis were the most preferred nitrite users.

Table 1 reports drug use characteristics: 26% (n/N = 27/105) revealed weekly practices, 8% (n/N = 6/68) said they engaged in slamming, 64% (n/N = 67/105) sometimes had group sex, 10% (n/N = 10/104) did not remember having had sober sex in the past 6 months, and 41% (n/N = 43/104) had had a negative experience such as a loss of consciousness, hallucinations, paranoia, or non-consensual sex. Table 2 outlines the main reasons for concerns and needs related to chemsex practices: 49% of participants disclosed being worried about chemsex use, and 18% said they needed help.

**Table 1:** Drug use characterization

	n (%)
Route of drug administration	
Oral (N = 98)	83 (85%)
Inhaled (N = 93)	78 (84%)
Sniffed (N = 85)	55 (65%)
Sublingual (N = 67)	16 (24%)
Rectal (N = 69)	10 (14%)
Injecting or *slamming* (N = 68)	6 (9%)
Sharing injection material if *slamming* (N = 3)^1^	3 (100%)
Use of sterile material if *slamming* (N = 6)	6 (100%)
Frequency of drug use	
Every day (N = 105)	7 (7%)
Every week (N = 105)	27 (26%)
Every month (N = 105)	32 (30%)
Less than once a month (N = 105)	39 (37%)
Number of drugs used	
1 drug (N = 103)	40 (39%)
2 drugs (N = 103)	41 (40%)
Poly drug use^2^ (N = 103)	22 (21%)
Other factors related to drug use	
Group sex (N = 105)	
No	30 (29%)
Sometimes	67(64%)
Usually	8 (8%)
Individuals who did not remember the last time they had sex without using drugs (N = 104)	8 (8%)
Individuals who had sober sex in the past 6 months (N = 104)	
Never	10 (10%)
Sometimes	42 (40%)
Always	52 (50%)
Individuals who had negative experiences (a loss of consciousness, hallucinations, paranoia, non-consensual sex) (N = 104)	
No	61 (59%)
Yes	21 (20%)
Sometimes	22 (21%)
Individuals who bring and consume their own drugs in chemsex sessions (N = 103)	
No	34(33%)
Yes	45 (44%)
Sometimes	24 (23%)
Individuals who consume drugs offered by other people (N = 104)	
No	14 (13%)
Yes	26 (25%)
Sometimes	64(62%)

^1^Three individuals practicing *slamming* did not answer the question

^2^*Consumption of* ≥ *3 drugs*

**Table 2:** Concerns and reasons for requesting help

	n (%)
Concern	
Concern about the use of drugs in a sexual context (N = 104)	51 (49%)
Concerns about drug use (N = 49)	42 (86%)
Concerns about sexuality (N = 48)	38(79%)
Concerns about STIs (N = 49)	44(90%)
Need for help	
Do you think you need help? (N = 105)	26 (25%)
In relation to drug use (N = 24)	22 (92%)
In relation to sexuality (N = 24)	17(71%)
In relation to possible STIs (N = 24)	21 (88%)

**Fig. 3:**
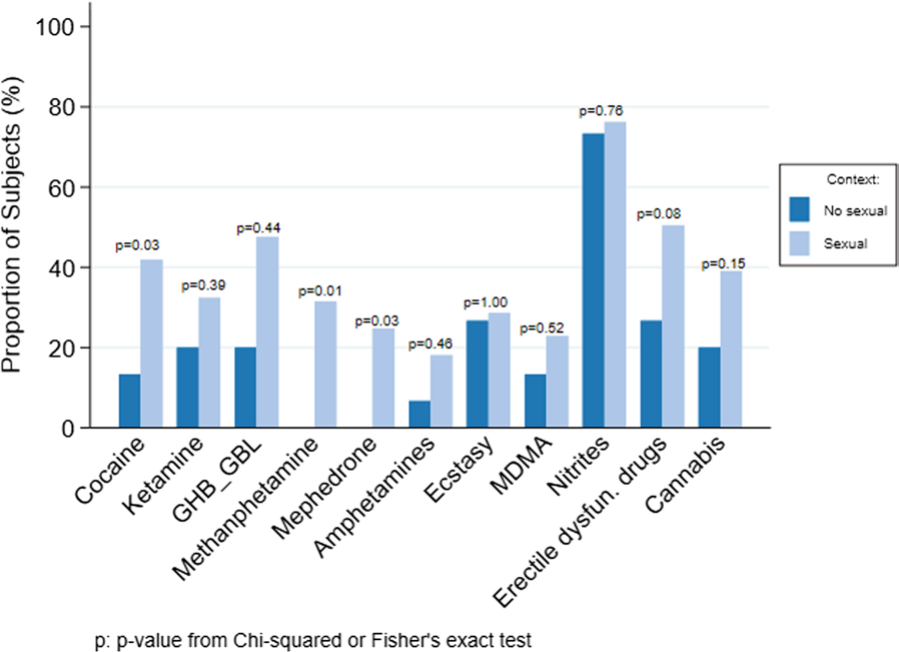
Drug differences between chemsex practices and drug use in a non-sexual context

When compared to chemsex practices versus non-sexual context drug use, we found that in terms of consumption, methamphetamine constituted 31% vs. 0% (χ^2^ = 6.5, p = 0.0108), mephedrone comprised 25% vs. 0% (χ^2^ = 4.7, p = 0.0294), GHB/GBL made up 48% vs. 20% (χ^2^ = 4.06, p = 0.0439) (see Fig. 3). Certain sexual behaviors were more prevalent in chemsex users, such as open relationships (82% vs. 42%, p = 0.0213), group sex (76% vs. 41%, χ^2^ = 21.1, p ≤ 0.0001), double penetration (26% vs. 5%, χ^2^ = 11.5, p = 0.0007), and fisting (23% vs. 5%, χ^2^ = 9.6, p = 0.0020). We did not find any differences in STIs between the two groups, specifically positivity for STI PCR samples (32% vs. 27%, χ^2^ = 0.4, p = 0.5433) and in VDRL values (11% vs. 6%, χ^2^ = 1.8, p = 0.416).

## Discussion

Substance use among our PrEP cohort was high (89%), especially chemsex practices exceeding 60%. This rate is considerably higher than those reported in other European and American PrEP cohorts, which are over 30 to 40%. In the PROUD study in England, it was 38.5% [23]. Hoornenborg et al. in Amsterdam, reported a rate of 41% of Chemsex practice with 3 or more partners [19], while 30% was found in a sub-study of the Ipergay trial, performed in France and Canada [18]. Flores Anato JL et al. discovered 24% in their Canadian cohort recently published [24]. Differences between studies may be due to the use of heterogeneous definitions of chemsex practices (some research groups limit the phenomenon to the use of GHB/GBL, mephedrone, and methamphetamine consumption), which may be influenced by local epidemiological factors. In Spain, Barcelona is the city with the highest prevalence of chemsex according to data collected in the EMIS 2017 survey [17].

Chemsex is a dynamic phenomenon but seems to be on rise in the global GBMSM population. This is also evidenced in our study, as more than half of the users disclosed having initiated chemsex practices in the past year. One out of 5 respondents reported weekly practices, highlighting a considerable profile of chemsex and PrEP users who engage in chemsex on a frequent basis. This fact may be a reflection of socialization, mainly the sexualized leisure found in the subgroup of GBMSM.

Polydrug consumption is almost twice as frequent among our PrEP users compared to other study groups. In the work of Flores Anato JL et al. 13% of users reported polysubstance use [24]. Moreover, 68% expressed using substances along with alcohol. We want to highlight this fact since the combination of depressant substances (such as alcohol with GHB/GBL or ketamine) may trigger a serious situation, which can range from a slight loss of consciousness to cardiorespiratory arrest [25].

In the present study, the most relevant drugs related to chemsex practices (compared to non-sexual drug consumption) were methamphetamine, mephedrone, cocaine, and GHB/GBL; the preferred combination was methamphetamine, GHB/GBL, nitrites, and erectile dysfunction drugs. These substances are the ones primarily related to chemsex worldwide [26, 27]. However, mephedrone consumption among our PrEP users was less common that in other cohorts [11, 28]. Moreover, in Spain, cocaine is still very prevalent, as well as in chemsex practices [29]. Hence, when defining chemsex, it is important (in addition to drugs) to take into account the geographic and cultural variability of the phenomenon.

Slamming was disclosed in 8% of the participants. This rate is higher than those observed in other PrEP cohorts: 2% in a Canadian study and 4% in a cohort from Amsterdam [24, 30]. The slamming prevalence reported in EMIS 2017 in Spain is also lower [17]. Injected drug use may be associated with negative consequences that health professionals should be aware of in order to ensure proper accompaniment, in addition to drug consumption counseling and harm reduction strategies [31].

Another relevant finding was the rate of sober sex; 10% of respondents disclosed not having had sober sex in the past 6 months, and 8% did not remember the last time they had had sober sex. Recent publications have described how chemsex users experience sex in a more impersonal way, whereas sober sex implies a more intimate and affective relationship. This may be a reflection of the potential difficulty of facing one’s own insecurities and anxieties when trying to have sober sex [32, 33]. More research to find solutions to these personal issues should be a priority for the comprehensive approach that these people deserve.

Our study is the first to assesses the consequences of chemsex in users taking PrEP related to sexual and psychological health; 41% of respondents expressed having had negative experiences in general (a loss of consciousness, hallucinations, paranoia, and non-consensual sex). Data available on this theme among chemsex GBMSM users are scarce; the Irish cohort reported a 23% loss of consciousness [34]. Further, MSM who engage in chemsex are more likely to disclose bad experiences and sexual contact without consent in the past year [35], and 19% of users have experienced non-consensual sexual practices [36]. These findings underline an important issue to address how consent is affected in chemsex.

Different researchers [37, 38] have already described the association of psychotic symptoms with previously mentioned drugs, especially with methamphetamine use. It is important for health care professionals to acquire skills to identify these symptoms and to establish good, rapid referral networks for psychiatric management.

Almost half of the participants expressed worries about chemsex practices, most of them due to consumption management, sexual issues, and the risk of contracting an STI. Approximately one-fifth felt they needed help with these aspects.

Despite the moderate rate of negative experiences, 59% of users enjoyed chemsex practices, similar to the proportions observed in other general GBMSM cohorts [34, 36]. The polarization of chemsex practices between those that are problematic or those that are non-problematic has been mentioned in the literature [39], but this binary point of view may have implicit limitations, such as offering support only when negative consequences are detected [40]. There is a recent perspective whereby chemsex is seen as a continuum of practices called the chemsex journey model [41]. Problematic chemsex is defined as subjectively experiencing one or more unwanted outcomes of a dynamic, contextualized process that consists of multiple stages. This approach suggests opportunities to support people at earlier stages of chemsex, rather than waiting until their use becomes problematic from their individual perspective.

This study has some limitations. We did not provide follow-up results since the study is limited to baseline characterization. The sample size is not as large as that of other general PrEP cohorts; however, we report specific data from chemsex practices that other cohorts do not. We did not ask specifically about negative experiences, but it would be interesting to know which ones have been the most frequent. Nor did we ask about the positive things that users experience, so these are complementary variables to add in future analyses.

## Conclusions

The prevalence of chemsex practices among our PrEP users is higher than that described in other cohorts, as is the rate of polycomsuption and slamming. Sexual practices at higher risk have been significantly described in our PrEP users engaged in chemsex. A baseline difficulty for sober sex and negative experiences related to the chemsex practice, have been reported by some participants. Almost one in four of the participants disclose concerns and need of help related to drug consumption management, issues related to sexuality, and STIs. These findings encompass the priority of an interdisciplinary and integral approach that includes substance information and risk management measures in the context of higher risk sexual practices.

Health professionals should offer a safe space, without judgment, where users may freely express their sexual experiences and feelings. This is why we believe that PrEP services teams should incorporate not only an STI expert, but also a drug expert psychiatrist or psychologist and a sexologist, and should work closely with the community and community entities, to offer, beyond addiction and risk, a fuller approach to enjoy a healthy sex life.
